# Medical News and Items

**Published:** 1875-11

**Authors:** 


### Medical News and Items.

The Editorial Management of the JOURNAL AND EXAMINER, take this opportunity of requesting physicians who are conducting clinics in the city, to make some provision for the regular reporting of their interesting cases in these columns. It is desired to make this Journal, as far as possible, a mirror of the clinical practice in the several hospitals of our city. If the gentlemen referred to, will appoint regular reporters of these clinical cases, and will accredit them to the editors, every effort will be made to afford them due representation.

College Openings.—The lectures in the Chicago Medical College for the Session of 1875–6 were inaugurated on the evening of October 4, by Prof. H. A. Johnson. The subject of the address delivered on this occasion was, the relations of the physical and spiritual universe to man and the duties of the educated physician resulting therefrom. The lecture was delivered with great animation and was heard with marked attention by a large number of students, professional gentlemen and ladies, who had assembled in consequence of the interest arising from the inaugural lecture of the course.

On the conclusion of the address the audience dispersed through the museum, laboratory and halls of the college, which were opened and lighted for their accommodation.

The Departments of Surgery, of Diseases of the Skin and of the Eye, Ear and Chest, connected with the South Side Dispensary, have been recently removed to commodious rooms in the basement of the College Building, which have been fitted up for that purpose, and furnished with apparatus for heating, lighting and providing hot and cold water for the attending physicians and surgeons.

The opening exercises of the winter session of Rush Colllege were held in the (temporary) college building, corner 18th and Arnold streets, on the evening of Sept. 29th. About two hundred students were assembled. An introductory prayer was offered by Rev. John Williamson, after which a brief address was delivered by Prof. Edwin Powell.

The facilities afforded by the Dispensary accommodations in the building of the Chicago Medical College, referred to above, enable the Faculty of that institution to give henceforth regular clinical lectures in their own amphitheatre. As heretofore, there will be also daily clinics in the gynecological rooms of the College.

The regular session of the Woman's Hospital College was begun on the morning of October 5th, with the regular didactic lectures.

Would it not be sensible for all colleges to follow the example of this, and omit any ceremony of opening the regular course of lectures?

CHANGES IN THE FACULTIES.—Since the close of the last winter lecture terms, several changes have occurred in the faculties of the colleges.

In Rush College, the chair of anatomy, made vacant by the retirement of Prof. R. L. Rea, has been filled by the election of Dr. Charles T. Parkes, the Demonstrator of Anatomy. Dr. Albert B. Strong, Lecturer on Materia Medica and Therapeutics in the Spring Faculty, has been elected Demonstrator. As Dr. Parkes was also Lecturer on Anatomy in the Spring Faculty, these changes make two vacancies in that body.

In the Woman's Hospital College, Prof. John Bartlett has been transferred from the chair of Diseases of Children to the associate chair of Practice of Medicine. Prof. Charles W. Earle has been transferred from the chair of Physiology to that of Diseases of Children; and Dr. Sarah Hackett Stevenson has been made professor of Physiology. Prof. A. H. Foster has resigned the chair of Surgery.

In the Chicago Medical College the chair of Hygiene has been made vacant by the resignation of Prof. Thos. Bevan. Prof. H. A. Johnson, having resigned the chair of Diseases of the Respiratory and Circulatory Organs, has been made a professor of Clinical Medicine.

Dr. D. A. K. Steele has been appointed one of the Assistant Surgeons of the Woman's Hospital of the State of Illinois.

